# Supplementary material for: A Comparative Study of Advanced Stationary Phases for Fast Liquid Chromatography Separation of Synthetic Food Colorants
Source: Molecules. 2018 Dec 15;23(12):3335. doi: 10.3390/molecules23123335 (PMC6321072; doi:10.3390/molecules23123335)
Supplement: Supplementary file 1 [file molecules-23-03335-s001.pdf]

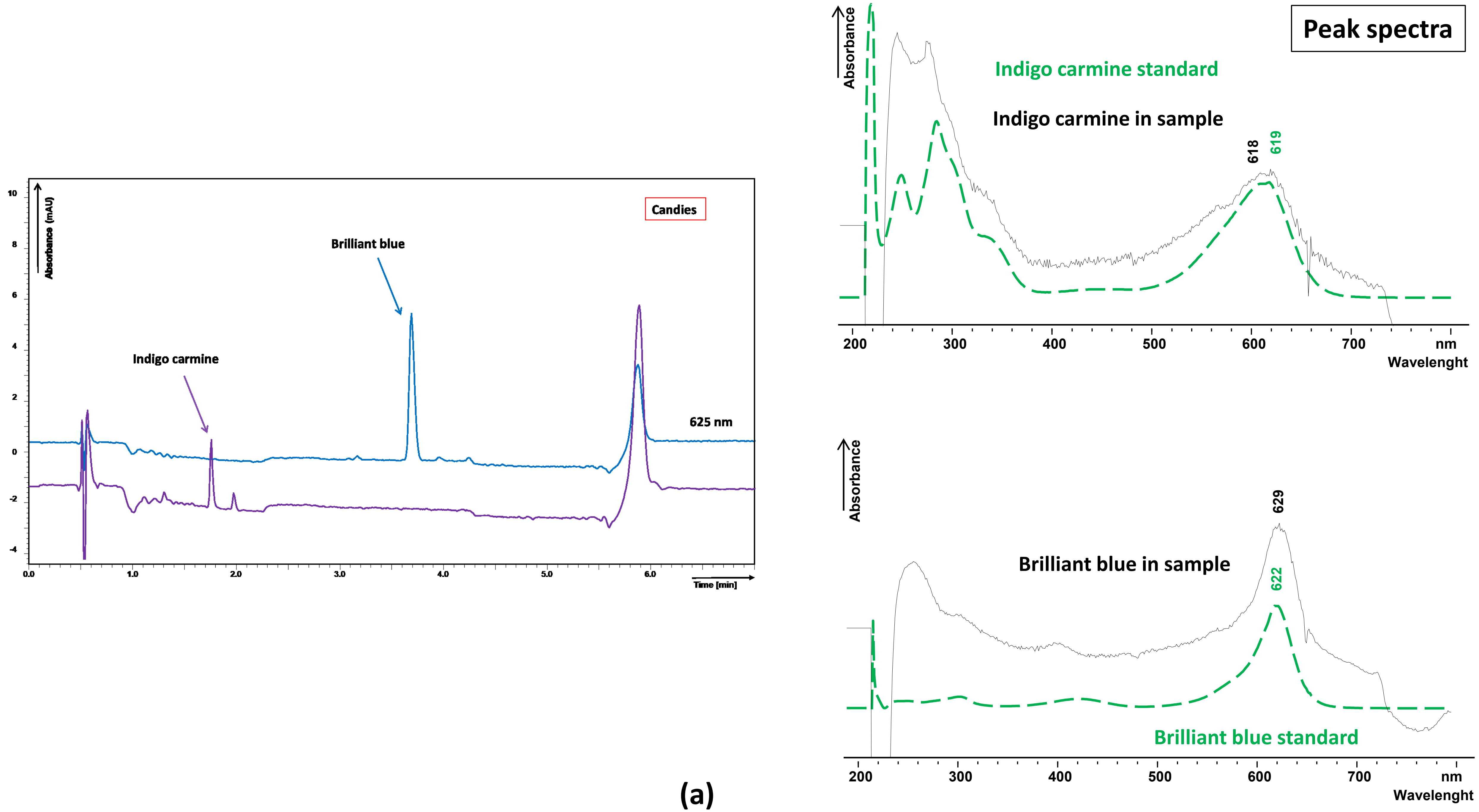
Absorbance (mAU)
10
8
6
4
2
0
-2
-4
Candies
Brilliant blue
Indigo carmine
625 nm
0.0
1.0
2.0
3.0
4.0
5.0
6.0
Time [min]
Peak spectra
Absorbance
Indigo carmine standard
Indigo carmine in sample
618
619
200
300
400
500
600
700
nm
Wavelenght
Absorbance
629
Brilliant blue in sample
622
Brilliant blue standard
200
300
400
500
600
700
nm
Wavelenght

(a)

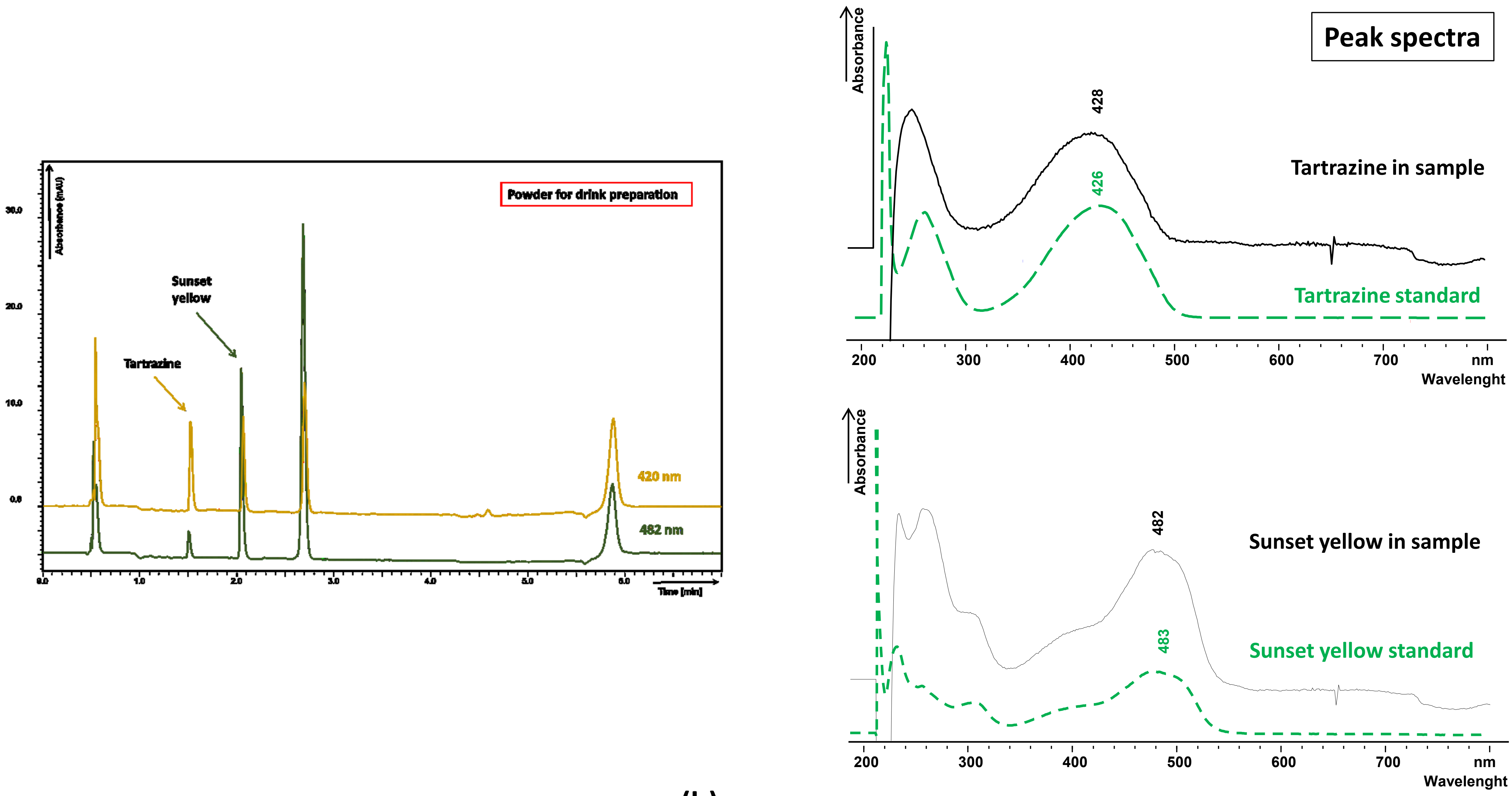

Powder for drink preparation
Absorbance (mAU)
Sunset yellow
Tartrazine
420 nm
482 nm
Time [min]
Peak spectra
Absorbance
428
Tartrazine in sample
426
Tartrazine standard
200
300
400
500
600
700
nm
Wavelenght
Absorbance
482
Sunset yellow in sample
483
Sunset yellow standard
200
300
400
500
600
700
nm
Wavelenght

(b)

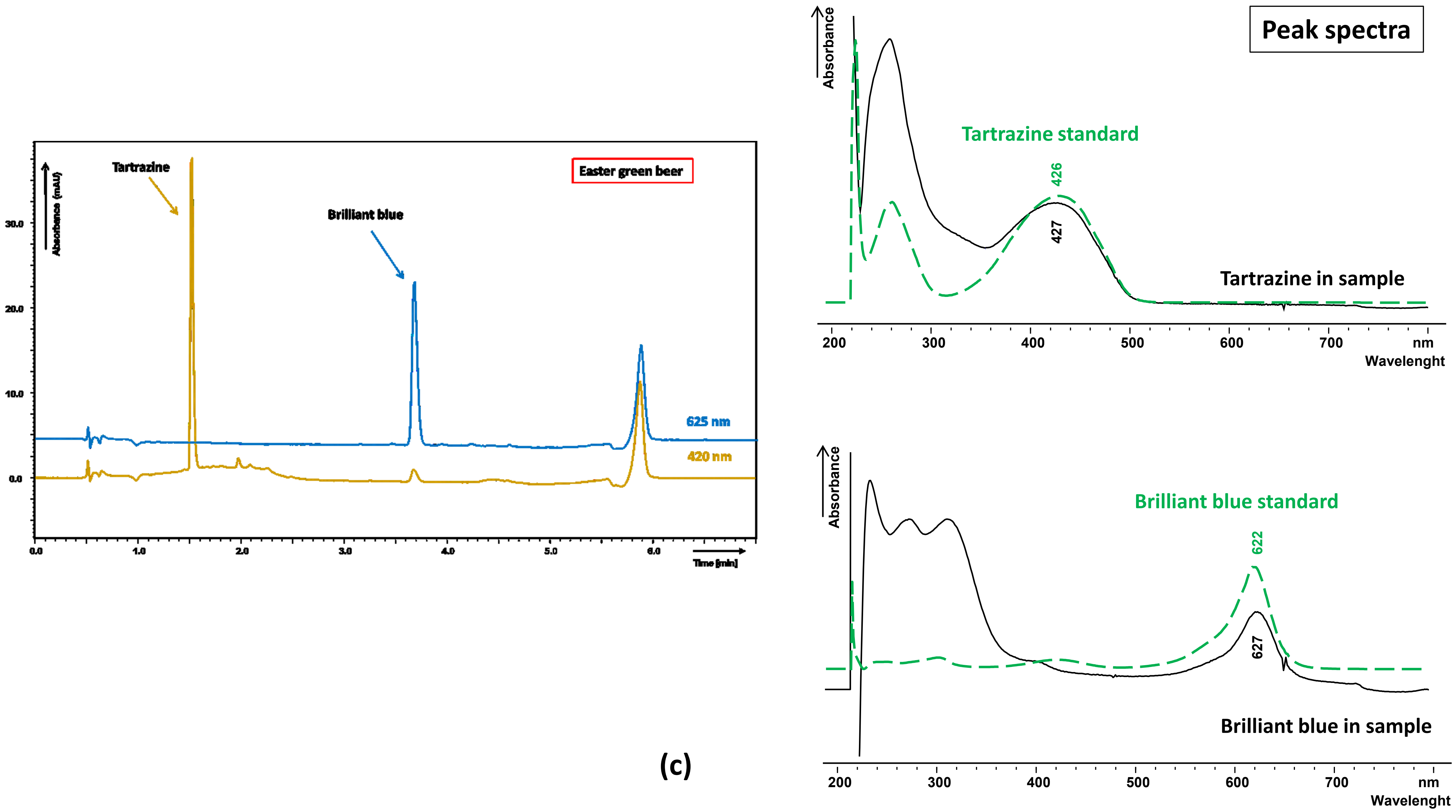

Absorbance (mAU)
Tartrazine
Brilliant blue
Easter green beer
30.0
20.0
10.0
0.0
625 nm
420 nm
0.0
1.0
2.0
3.0
4.0
5.0
6.0
Time [min]
Peak spectra
Absorbance
Tartrazine standard
426
427
Tartrazine in sample
200
300
400
500
600
700
nm
Wavelenght
Absorbance
Brilliant blue standard
622
627
Brilliant blue in sample
200
300
400
500
600
700
nm
Wavelenght

(c)

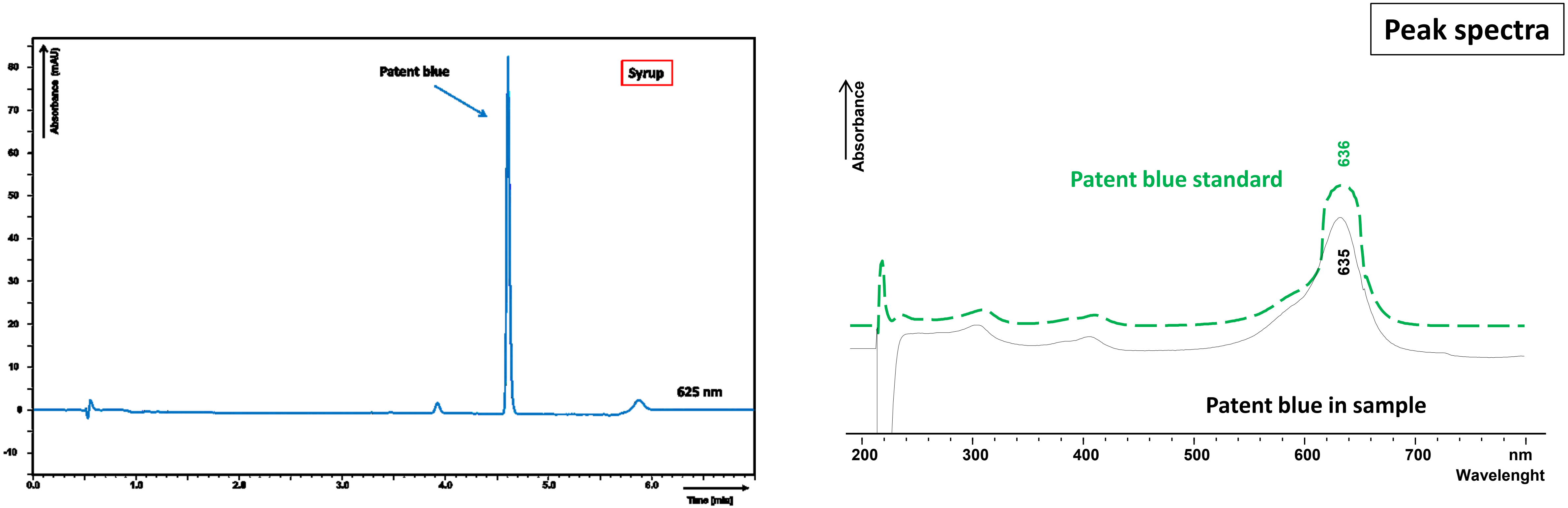
Absorbance (mAU)
80
70
60
50
40
30
20
10
0
-10
Patent blue
Syrup
625 nm
0.0
1.0
2.0
3.0
4.0
5.0
6.0
Time [min]
Peak spectra
Absorbance
636
Patent blue standard
635
Patent blue in sample
200
300
400
500
600
700
nm
Wavelenght

(d)

Figure S1: Comparison of UV/VIS spectra of detected peaks in samples with the spectra of standard solution for confirmation of their indentity. (a) candy, (b) powder for drink preparation, (c) colored beer, (d) syrup.
